# Lab values in neonates with hypoxic ischemic encephalopathy over time during and after therapeutic hypothermia

**DOI:** 10.3389/fped.2026.1743749

**Published:** 2026-03-12

**Authors:** Michael Elias, Nikolay Bliznyuk, Daphna Yasova Barbeau, Sarah Sukumar, Juan Carlos Roig, Dhanashree Rajderkar, Livia Sura, James L. Wynn, Michael D. Weiss

**Affiliations:** 1 Department of Pediatrics, University of Florida, Gainesville, FL, United States; 2 Departments of Agricultural and Biological Engineering, Biostatistics and Statistics, University of Florida, Gainesville, FL, United States; 3 Envision Physician Services, Davie, FL, United States; 4 Department of Radiology, Division of Pediatric Radiology, University of Florida, Gainesville, FL, United States

**Keywords:** encephalopathy, hypoxic-ischemic, laboratory biomarkers, neonatal brain injury, therapeutic hypothermia

## Abstract

**Introduction:**

Hypoxic-ischemic encephalopathy (HIE) remains a leading cause of neonatal neurological injury, and therapeutic hypothermia is the established treatment shown to reduce brain injury in neonates with moderate to severe HIE. The systemic laboratory response to hypoxic-ischemic injury and its relationship to brain injury severity are not fully understood.

**Methods:**

This retrospective cohort included 152 neonates born at a gestational age of 35 weeks or greater who met criteria for therapeutic hypothermia for HIE at UF Health Shands Children's Hospital between 2012 and 2024. Laboratory data were collected at seven time intervals from birth through rewarming and analyzed using linear mixed-effects models to characterize temporal trends. Temporal analyses revealed changes across metabolic, hepatic, and coagulation biomarkers during and after therapeutic hypothermia, with several values demonstrating significant variation at specific time points. Neonates were classified by sentinel event status (definite, probable, or none), and temporal trends demonstrated differences between the groups. The laboratory values were correlated with magnetic resonance imaging (MRI) injury severity using the Weeke scoring system.

**Results:**

Early metabolic derangements, including lower pH and more negative base deficit values, were significantly associated with higher MRI injury scores across all regions, including gray matter, white matter, and the cerebellum. Machine-learning models that integrate a combination of early laboratory timepoints improve the prediction of MRI-defined injury, with the best performance achieved using pH at T1, pCO₂ at T1, and lactate at T3 (adjusted *R*² = 0.47).

**Conclusion:**

These findings demonstrate temporal laboratory trajectories during and after therapeutic hypothermia, supporting the prognostic utility of serial biomarkers and machine-learning-based modeling in neonatal HIE.

## 1 Introduction

Hypoxic-ischemic encephalopathy (HIE) remains a significant cause of neurological morbidity and mortality in neonates. Despite extensive research, many aspects of its pathophysiology, treatment, and long-term outcomes remain poorly understood (1). The estimated incidence of HIE is approximately 1.5 per 1,000 live births (2). Currently, therapeutic hypothermia (TH) is the gold standard treatment to mitigate hypoxic-ischemic injury in this population. The therapeutic window for initiating TH is within the first 6 h of life, a critical period aimed at reducing inflammation and apoptosis pathways that contribute to secondary brain injury (3).

Supportive care during therapeutic hypothermia (TH) remains a cornerstone of management for neonates with hypoxic-ischemic encephalopathy (HIE), even as research continues to explore innovative adjunctive therapies to improve outcomes.

Hypoxia-ischemia can result in multi-organ injury, as reflected by laboratory markers of end-organ ischemia, including elevated liver enzymes, cardiac enzymes, lactate levels, and abnormalities in coagulation studies. These laboratory values are closely monitored and trended throughout the course of illness to guide clinical management and assess disease progression. Emerging evidence highlights that factors such as hypocapnia, hyperoxia, extreme fluctuations in blood pressure and glucose are associated with worse outcomes in this population (1, 4–8). However, the temporal patterns and thresholds of laboratory values that reliably predict poor prognosis have not been well defined in the literature. Identifying these patterns may offer new insights into optimizing supportive care and improving prognostic accuracy in neonates undergoing TH.

This study aims to provide a deeper understanding of the expected temporal patterns of laboratory values in neonates undergoing therapeutic hypothermia (TH). By identifying these patterns, clinicians may be able to recognize infants who deviate from the expected trajectory earlier in the course of treatment, prompting further evaluation for alternative or additional diagnoses. Furthermore, we hypothesize that the severity of brain injury in neonates with HIE, as measured by MRI, will correlate with abnormal laboratory values over time. These differences may serve as valuable clinical indicators to guide and optimize the management of these critically ill patients.

## 2 Materials and methods

### 2.1 Patient populations

This study was approved by the University of Florida Institutional Review Board (IRB). All participants were enrolled at a single site, UF Health Shands Children's Hospital, between 2012 and 2024.

### 2.2 HIE population inclusion and exclusion criteria

A total of 152 neonates with hypoxic-ischemic encephalopathy (HIE) who met eligibility criteria for therapeutic hypothermia (TH) were recruited. All infants underwent standardized whole-body therapeutic hypothermia according to institutional protocol. The entry criteria for hypothermia included a gestational age of 35 weeks or greater, a birth weight of 1.8 kg or greater and less than or equal to 6 h of age at the time of evaluation. Evidence of encephalopathy was defined by the presence of seizures or abnormalities on a modified Sarnat examination, which assessed level of consciousness, spontaneous activity, posture and tone, primitive reflexes (suck and Moro), and autonomic function, including pupils, heart rate, and respirations. Hypoxic-ischemic injury was diagnosed based on one or more of the following criteria: arterial pH ≤7.0 and/or base deficit ≥16 mmol/L, arterial pH between 7.01 and 7.15 and/or base deficit between 10 and 15.9 mmol/L, or the absence of a blood gas with evidence of an acute perinatal event such as cord prolapse, fetal heart rate decelerations, or uterine rupture. Exclusion criteria included the presence of known chromosomal abnormalities, significant intracranial hemorrhage, and severe intrauterine growth restriction (IUGR). Therapeutic hypothermia was administered using the CritiCool™ blanket device (Mennen Medical Corp., Feasterville–Trevose, PA) with a target temperature of 33.5 °C for 72 h. All neonates received sedation during therapeutic hypothermia using a dexmedetomidine infusion, a fentanyl infusion, or a combination of both.

### 2.3 Laboratory data collection

Data were collected at predefined time points during routine clinical monitoring for each infant. These time points included pre-cooling (T1: 0–6 h), during cooling (T2: 6–12 h, T3: 12–24 h, T4: 24–48 h, and T5: 48–72 h), and post-cooling (T6: 72–96 h and T7: >96 h). To ensure comprehensive data collection and analysis, an Integrated Data Repository (IDR) was established in collaboration with the University of Florida Clinical and Translational Science Institute (UF CTSI). The IDR incorporated clinical data extracted from the electronic health record (EHR), encompassing both structured and unstructured data elements. This dataset included subject demographics, clinical parameters, and laboratory values. If multiple laboratory values were available within a given time interval, the first recorded value was used for T1, T2, and T3. For subsequent timepoints, when more than one value was available, the value closest to the midpoint of the interval was selected.

For the temporal trend analysis, the entire cohort was examined. A secondary analysis then stratified the cohort into three groups. Maternal-neonatal dyad chart reviews classified each infant according to the timing and nature of the hypoxic-ischemic insult. The group-specific criteria were defined as follows:

**Group 1:** Neonates with a history of definite sentinel events, such as cord prolapse, uterine rupture, placental abruption, placenta previa, maternal hypotension following trauma or epidural analgesia, and shoulder dystocia. The presence of these events during the peripartum period indicated that the hypoxic-ischemic injury occurred at birth.
**Group 2:** Neonates with a history of probable sentinel events, including prolonged decelerations without a clear etiology, cord compression, or a true knot, suggesting that the hypoxic-ischemic injury occurred within a few hours before birth.
**Group 3:** Neonates with no known history of sentinel events who experienced hypoxia-ischemia and underwent therapeutic hypothermia. These neonates may have suffered from chronic or acute-on-chronic hypoxic-ischemic injury.

Two physicians independently reviewed the charts to classify neonates into one of the three groups. When available, prenatal monitoring strips were analyzed by a board-certified obstetrician to confirm the timing of injury. Fetal monitoring strips were available only for inborn neonates (*n* = 13). The time of hypoxic-ischemic insult was determined based on the onset of prolonged decelerations or the progressive depth of variable or late decelerations.

### 2.4 MRI scoring

MRI scans were performed at 3–6 days of age following rewarming (*n* = 108), at 7–12 days of age (*n* = 35; delayed due to clinical instability at 3–6 days), and at 20–53 days of age (*n* = 6; delayed due to prolonged clinical instability). Neonates were imaged on a Siemens Magnetom Verio 3T scanner (Siemens, Malvern, PA) (*n* = 91) or a Siemens Magnetom Avanto 1.5T (Siemens, Malvern, PA) (*n* = 60) at UF Health Gainesville. A single blinded subspecialty board-certified neuroradiologist with over 10 years of experience in neonatal imaging interpreted all the MRI images using the Weeke scoring system (9). The Weeke scoring system evaluates brain injury across three regions: deep gray matter, white matter/cortex, and the cerebellum, with an additional subscore assessing the presence of intraventricular hemorrhage (IVH), subdural hemorrhage (SDH), and cerebral sinovenous thrombosis (CSVT). Each anatomical region is systematically scored based on the extent and distribution of injury. The total score is calculated by summing the scores from the gray matter, white matter/cortex, cerebellum, and additional categories, with a maximum score of 55. If 1H-MRS data are available, abnormalities in the basal ganglia and thalamus, such as reduced N-acetyl aspartate (NAA) or elevated lactate peaks, are incorporated into the gray matter subscore, increasing the total score to a maximum of 57.

### 2.5 Statistical analysis

#### 2.5.1 Data analysis for individual biomarkers

Exploratory analysis was carried out using box plots that correspond to one-way and two-way ANOVA models for biomarkers as the outcome (response) variable and the main effects of timepoint, the sentinel event group, and their interaction. Normal-theory linear mixed models (LMMs) with subject-specific random effects were used to test the overall significance of the trends over time and the interaction with the sentinel event group (10). Given the exploratory nature of this study and the large number of hypotheses evaluated, no formal adjustment for multiple comparisons was applied. As expected of biological data in a repeated measures experiment, the analysis highlighted strong subject-specific effects. To mitigate the effect of potential outliers (on inflating the variability) present in several biomarkers, statistical analyses were performed and compared after “trimming”, i.e., removing q% of observations from both tails of the distribution for *q* = 0, 1 and 2.5; e.g., for *q* = 2.5, the analysis used 95% of the (non-extreme) data for each biomarker. Subsequently, individual MRI scores were correlated with biomarker measurements at individual timepoints using simple linear regressions and reported as coefficient estimates and *p*-values. Finally, individual MRI scores were thresholded as mild (MRI ≤ 2) and severe (MRI > 2); and pairwise *t*-tests were performed for each biomarker-by-timepoint to identify statistically significant differences between the severity groups.

We explored the value of individual biomarkers from each of the first three timepoints for the purpose of early detection (prediction) of the MRI injury severity group using logistic regression. The results were summarized using standard metrics of the binary classifier performance, including misclassification error rate (MER), sensitivity, specificity, ROC curves and the area under the ROC curve (AUC). Only observed data without imputation were used for all analyses in this subsection.

#### 2.5.2 Multivariate predictive modeling of total MRI score

Additionally, we investigated potential improvements from *jointly* using multiple biomarkers from the first three timepoints to predict the total MRI numerical score, a task that is complicated by considerable biomarker data missingness. To ensure an honest predictive performance evaluation, we used the following data-analytic strategy to proceed: (i) we prescreened the 54 predictors (corresponding to the 18 biomarkers at time points T1–T3) and retained those with 2/3 (66.67%) or higher proportion of non-missingness (leading to approximately 100 observed measurements on each of 22 predictors), (ii) we imputed the missing predictor values using the multivariate imputation via chained equations (11) algorithm implemented in R exclusively using observed values of the *predictors* (i.e., without using the MRI score response that one targets to predict); (iii) we used five-fold block cross-validation for variable selection and training, where four folds were used for variable selection (using Bayesian Information Criterion, BIC) and the fifth fold was used for performance assessment (using adjusted *R*^2^). Here, “blocks” correspond to individual subjects' MRI value and the corresponding predictors (12). Imputation allows one to use a greater number of observations (training data points) and a greater number of predictors at the expense of making the covariates less informative, due to replacement of missing values with noisy or biased imputed values; however, the uninformative predictors are subsequently eliminated through variable selection. Lastly, we compared the performance of best models with imputed covariates with that using the top 2–3 predictors without imputation; this dataset had fewer observations due to biomarker missingness but the predictors were more informative due to the lack of “signal dilution” through the imputation. We further validated our findings by formally checking whether the predictor missingness was informative, by testing statistically (using Kolmogorov–Smirnov test and Kruskal–Wallis) for distributional consistency of the response and the (best) selected predictors across subjects that had complete data (*n* = 95; “complete group”) and the “partially observed” group (*n* = 56, where at least one predictor value was missing), which did not reveal statistically significant differences across the two groups (discussed further in the Section 4).

Our evaluation strategy and predictive performance results are conservative as they explicitly aim at avoiding “over-optimism”, and one would expect better practical performance in the situations where the identified covariates are fully observed or, at least, contain less missing values than in our dataset.

## 3 Results

### 3.1 Demographics

A total of 152 neonates with HIE were enrolled in the study (Table 1). Of these, 55% were male and 45% were female. The mean (± SD) gestational age at birth was 38 ± 2 weeks, and the mean birth weight was 3,214 ± 727 grams. The mean Apgar scores at 1, 5, and 10 min were 2 ± 2, 4 ± 2, and 5 ± 2, respectively. Cesarean section was the mode of delivery in 91 infants (60%), and 89 infants (58%) were outborn. The mean umbilical cord arterial pH was 7.03 ± 0.18, with a base deficit of −14 ± 6. Initial postnatal blood gas values demonstrated a mean pH of 7.21 ± 0.13, base deficit of −12 ± 5, and lactate level of 10 ± 6 mmol/L. The median time to MRI was 4 days (IQR: 4–6), and the median hospital length of stay was 6.5 days (IQR: 3–10) (Table 1).

**Table 1:** Infant characteristics at enrollment.

Infant characteristics at enrollment	NE (*n* = 152)
Sex (%)	
Female	45
Male	55
Gestational Age in weeks (mea*n* ± SD)	38 ± 2
Birth weight in grams (mea*n* ± SD)	3,214 ± 727
Apgar score at 1 min (mean ± SD)	2 ± 2
Apgar score at 5 min (mean ± SD)	4 ± 2
Apgar score at 10 min (mean ± SD)	5 ± 2
C-Section delivery *n* (%)	91 (60%)
Outborn	89 (58%)
^a^Respiratory support *n* (%)	89 (%)
^a^Ventilator *n* (%)	117 (77%)
CPAP *n* (%)	13 (9%)
^a^High Flow Nasal Cannula *n* (%)	5 (3%)
^a^Inotropic support *n* (%)	75 (49%)
Platelet transfusion *n* (%)	47 (31%)
Fresh frozen plasma transfusion *n* (%)	85 (56%)
Cryoprecipitate transfusion *n* (%)	27 (18%)
^a^History of seizures *n* (%)	53 (35%)
SARNAT score II *n* (%)	70 (46%)
^a^SARNAT score III *n* (%)	51 (33%)
Umbilical cord arterial pH (mean ± SD)	7.03 ± 0.18
Umbilical cord arterial Base Deficit (mean ± SD)	−14 ± 6
Initial pH^a^^,^^b^ (mean ± SD)	7.21 ± 0.13
Initial Base Deficit^a^^,^^b^ (mean ± SD)	−12 ± 5
Initial Lactate^a^ (mean ± SD)	10 ± 6
Time to MRI [median (IQR)]	4 days (IQR: 4–6 days)
Length of stay [median (IQR)]	6.5 days (IQR: 3–10 days)

^a^ Represent lab values upon admission to the NICU. Includes transported patients.

^b^ Arterial values (*n* = 66).

At enrollment, 117 infants (77%) required mechanical ventilation, 13 (9%) were on continuous positive airway pressure (CPAP), and 5 (3%) received high-flow nasal cannula. Inotropic support was administered to 75 infants (49%), and 53 (35%) had a clinical history of seizures. Based on Sarnat staging, 70 infants (46%) had stage II encephalopathy and 51 (33%) had stage III.

Among the 152 infants with HIE enrolled in the study, detailed birth event data were available for 135 infants. These infants were classified into Group 1 (*n* = 52), Group 2 (*n* = 20), and Group 3 (*n* = 63) based on clinical criteria (Table 2). The mean birth weights were similar across groups: 3,171 ± 731 g in Group 1, 3,251 ± 589 g in Group 2, and 3,200 ± 699 g in Group 3. The proportion of male infants was 47%, 55%, and 54% in Groups 1, 2, and 3, respectively. The mean gestational age was comparable between groups, ranging from 38.2 to 38.7 weeks. Apgar scores at 1, 5, and 10 min were low in all groups, with means of 2 ± 2, 4 ± 3, and 5 ± 2–3, respectively.

**Table 2:** Maternal–neonatal and clinical characteristics by sentinel event category.

Characteristic	Group 1 (*N* = 52)	Group 2 (*N* = 20)	Group 3 (*N* = 63)
Birth weight in grams (mean ± SD)	3,171 ± 731	3,251 ± 589	3,200 ± 699
Sex (%)			
Male	47%	55%	54%
Female	53%	45%	46%
Gestational Age in weeks (mean ± SD)	38.2 ± 2.6	38.7 ± 2	38.6 ± 1.8
Apgar score at 1sminute (mean ± SD)	2 ± 2	2 ± 2	2 ± 2
Apgar score at 5 min (mean ± SD)	4 ± 3	4 ± 3	4 ± 2
Apgar score at 10 min (mean ± SD)	5 ± 3	4 ± 3	5 ± 2
Outborn	60.7%	65%	59%
C-Section delivery *n* (%)	66.7%	35%	59%
Respiratory support *n* (%)	45 (87%)	18 (90%)	58 (92%)
Ventilator *n* (%)	37 (71%)	3 (15%)	55 (87%)
CPAP *n* (%)	6 (12%)	3 (15%)	2 (3%)
High Flow Nasal Cannula *n* (%)	2 (4%)	2 (10%)	1 (2%)
Inotropic support *n* (%)	23 (44%)	8 (40%)	34 (54%)
History of seizures *n* (%)	21 (40%)	5 (25%)	29 (46%)
SARNAT score II *n* (%)	24 (46%)	8 (40%)	32 (51%)
SARNAT score III *n* (%)	15 (29%)	6 (30%)	21 (33%)
Cord pH	6.91 ± 0.21	6.8 ± 0.16	6.98 ± 0.18
Cord Base Deficit	19.1 ± 9.5	18.4 ± 5.16	15.4 ± 6.27
Initial pH (mean ± SD)	7.16 ± 0.17	7.14 ± 0.16	7.13 ± 0.17
Initial Base Deficit (mean ± SD)	14.7 ± 6.49	15.2 ± 6.02	18.82 ± 6.27
Initial Lactate (mean ± SD)	9.8 ± 6	8.7 ± 5	11 ± 6
Time to MRI [median (IQR)]	4 days (IQR: 4–6.5 days)	5 days (IQR: 4–10 days)	4 days (IQR: 4–6 days)
Length of stay [median (IQR)]	9 days (IQR: 7–18.5 days)	8 days (IQR: 2.5–9 days)	15 days (IQR: 7–26.75 days)

Outborn status was common, with 61% of Group 1, 65% of Group 2, and 59% of Group 3 infants transferred from outside facilities. Cesarean delivery occurred most frequently in Group 1 (67%) and least in Group 2 (35%). Sarnat stage II was observed in approximately half of each group, while Sarnat stage III was noted in 29%–33% of infants. Cord pH was lowest in Group 2 (6.80 ± 0.16) and highest in Group 3 (6.98 ± 0.18), with corresponding base deficits of 19.1 ± 9.5, 18.4 ± 5.2, and 15.4 ± 6.3 in Groups 1, 2, and 3, respectively. Initial postnatal blood gas values showed mean pH values ranging from 7.13 to 7.16 and lactate levels of 8.7 to 11 mmol/L.

The majority of infants required respiratory support, with mechanical ventilation used in 71%–87% of cases across groups. Inotropic support was administered to 44% of Group 1, 40% of Group 2, and 54% of Group 3 infants. A history of clinical seizures was reported in 40%, 25%, and 46% of Groups 1, 2, and 3, respectively.

The median time to MRI was 4–5 days across groups. Median length of stay varied, with Group 3 infants having the longest hospitalizations [15 days (IQR: 7–26.8)] compared to 9 days [IQR: 7–18.5] in Group 1 and 8 days [IQR: 2.5–9] in Group 2 (Table 2).

### 3.2 CO2 Laboratory trends over time

#### 3.2.1 Laboratory trends over time in the entire cohort

**Figure 1:**
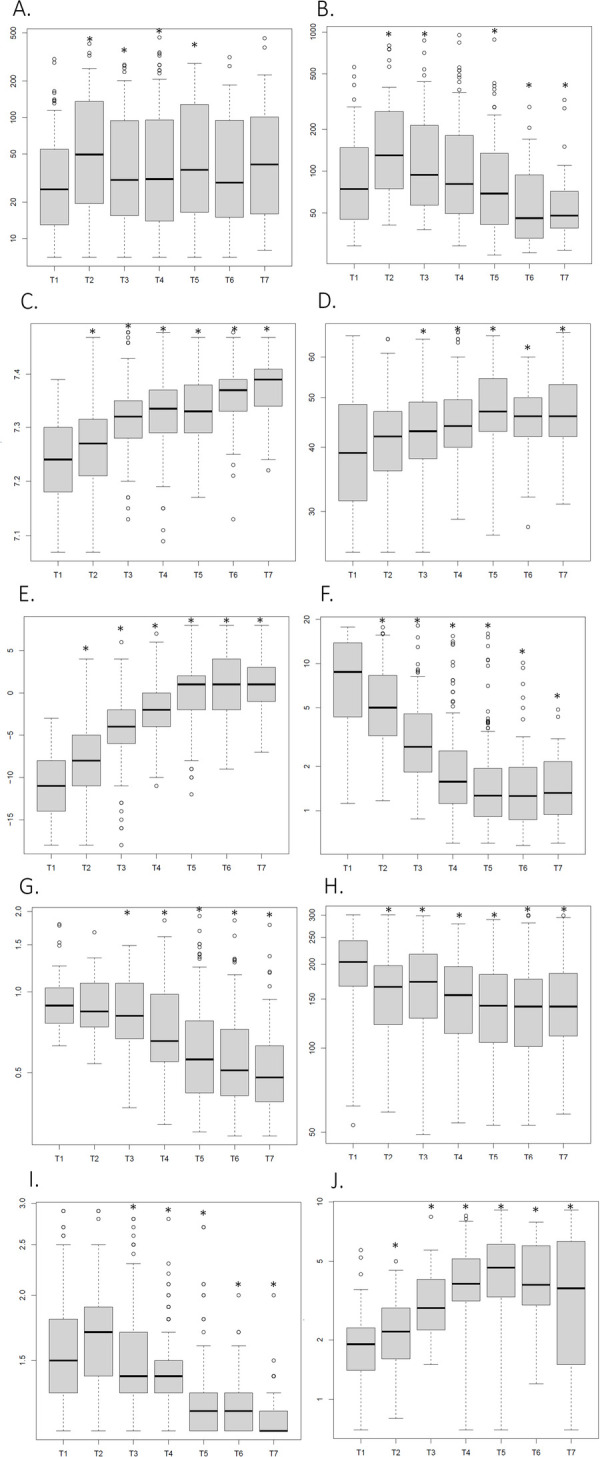
Temporal changes in laboratory biomarkers in neonates with hypoxic-ischemic encephalopathy treated with therapeutic hypothermia. Boxplots illustrate trends in **(A)** alanine aminotransferase (ALT)(y = U/L), **(B)** aspartate aminotransferase (AST)(y = U/L), **(C)** pH, **(D)** partial pressure of carbon dioxide (pCO_2_)(y = mmHg), **(E)** base deficit (BD)(y = mEq/L), **(F)** lactate (y = mmol/L), **(G)** creatinine (y = mg/dL), **(H)** platelets(y = ×10^9^/L), **(I)** prothrombin time–international normalized ratio (PT-INR, ratio), and **(J)** total bilirubin (y = mg/dL) across seven predefined time points (T1: 0–6 h, T2: 6–12 h, T3: 12–24 h, T4: 24–48 h, T5: 48–72 h, T6: 72–96 h, T7: >96 h of life), spanning the cooling, rewarming, and early post-therapy periods. Each box represents the interquartile range (IQR), with the horizontal line indicating the median. Whiskers extend to 1.5× IQR, and outliers are shown as individual points. A trimming parameter (q.trim = 0.025) was applied to reduce the influence of extreme values for visualization purposes.

In this section, we report the changes in systemic biomarker concentrations over time for the entire cohort (Figure 1). Alanine transaminase (ALT, U/L) concentrations peaked at T2 (median 50), relative to the baseline at T1 (median 25) (*p* < 0.05)(Figure 1A). Thereafter, ALT levels remained elevated from T3 to T5 compared with T1. Aspartate transaminase (AST, U/L) exhibited a similar but more pronounced trajectory, frequently elevated at T1 (median 75), peaking at T2 (median 130); *p* < 0.05), and remaining above baseline at T3 (median 94). From T5 to T7, AST levels declined significantly compared to T1 (*p* < 0.05)(Figure 1B).

Arterial pH was lowest at T1 (median 7.24) and increased steadily from T2 (median 7.27) through T7 (median 7.39) (*p* < 0.05)(Figure 1C). By T6, most infants had reached a pH of 7.40, with a subset exceeding this threshold and becoming alkalotic (pH > 7.40) (13–15). Similarly, arterial pCO2 (mmHg) was lowest at T1 (median 39)—exhibiting the greatest variability at that time point—and rose significantly at T3, T4, and T5 (*p* < 0.05)(Figure 1D). Overall, most pCO2 measurements remained within the normal range (35–45 mmHg) (13–15). Base deficit (mEq/L) followed a similar pattern, with the most negative values observed at T1 (median −11)(Figure 1E). Values improved significantly by T2 (median −8; *p* < 0.05) and T3 (*p* < 0.05), and normalized by T4 (median 2; *p* < 0.05), before shifting into a base excess from T5 through T7 (*p* < 0.05). In contrast to the trajectories observed for pH, pCO_2_, and base deficit, lactate concentrations (mmol/L) were highest at T1 (median 9) and declined significantly from T2 (median 5) through T7 (median 1; *p* < 0.05)(Figure 1F). By T4, most infants had lactate levels below 2 mmol/L, although a subset continued to exceed this threshold through T7.

Cortisol (µg/dL) levels exhibited wide variability, peaking at T1 and showing a significant decrease by T6 (*p* < 0.05). In parallel, serum creatinine (mg/dL) values steadily declined from T4 through T7(*p* < 0.05)(Figure 1G).

Creatine kinase (CK, U/L) levels peaked at T2 (*p* < 0.05), remained elevated from T3 to T4, and then declined steadily through T6, followed by an abrupt decrease at T7 (>96 h).

Platelet counts(x10^9^/L) were highest at T1 (median 204) and decreased significantly from T2 (median 166) through T7 (median 141) (*p* < 0.05)(Figure 1H). During therapeutic hypothermia, partial thromboplastin time (PTT, seconds) decreased progressively from T3 through T7 (*p* < 0.05). The prothrombin time/international normalized ratio (PT–INR, ratio) was highest at T1 and decreased significantly from T3 onward (*p* < 0.05)(Figure 1I). In contrast, fibrinogen (mg/dL) levels were lowest at T1 (median 185) but rose markedly from T3 to T7 compared with baseline (*p* < 0.05).

White blood cell counts (WBC, x10^9/L) were highest at T1 (median 16) and T2 (median 17), then decreased significantly from T3 through T5 (*p* < 0.05) and stabilized thereafter.

Total bilirubin (mg/dL) levels appeared less influenced by HIE and therapeutic hypothermia, following a largely physiologic pattern (Figure 1J). Beginning at T3 (median 3) (*p* < 0.05), total bilirubin rose from the T1 baseline (median 2), increasing steadily through T4 (median 4) (*p* < 0.05) and peaking at T5 (median 5) (*p* < 0.05). After T5, levels plateaued but remained significantly higher than at T1 (*p* < 0.05). Notably, in this cohort of infants undergoing therapeutic hypothermia, total bilirubin levels appeared lower than expected for term infants based on standard nomograms (Figure 1J).

#### 3.2.2 Laboratory trends over time in each of the 3 groups

**Figure 2:**
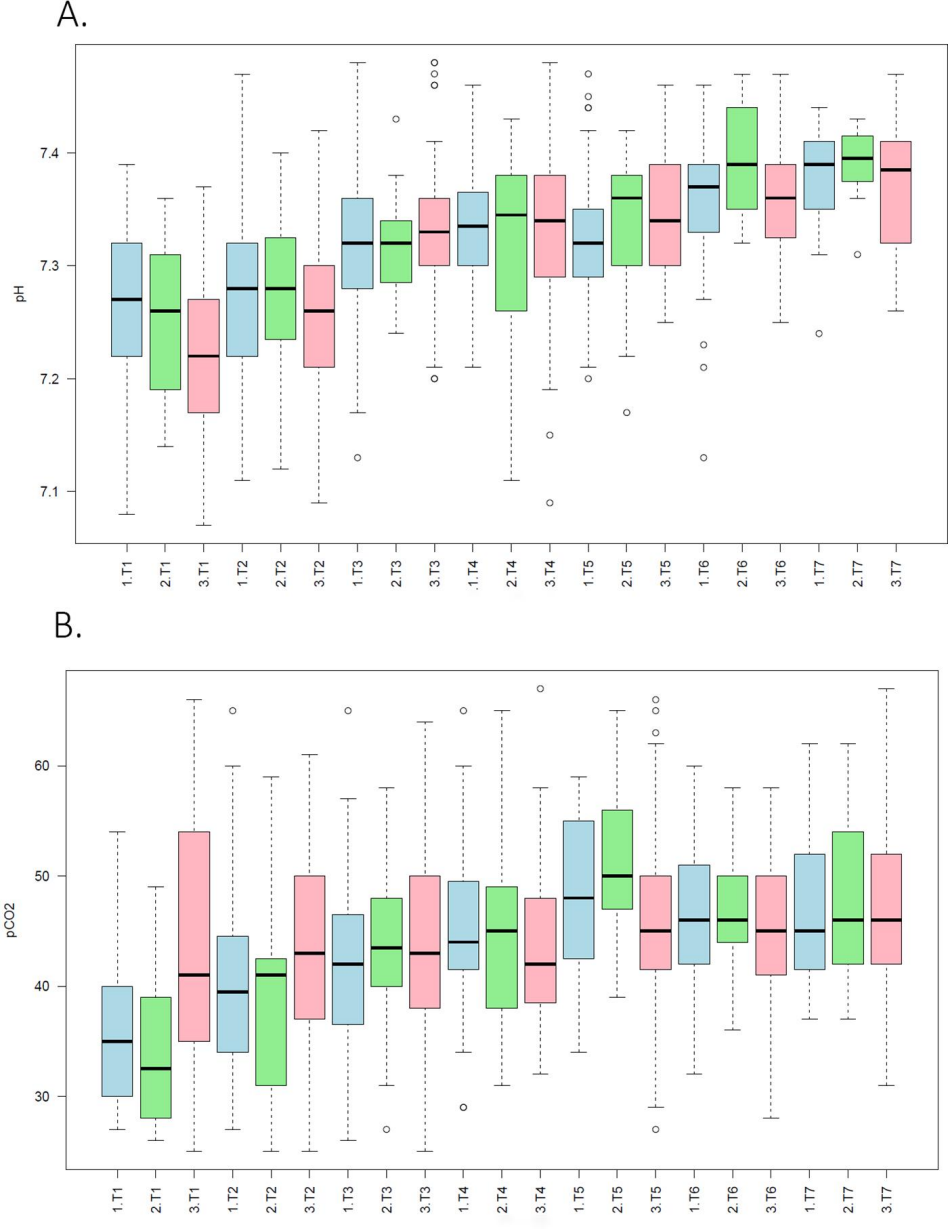
Stratified trends in arterial blood gas parameters by sentinel event status in neonates with hypoxic-ischemic encephalopathy. Boxplots display changes in **(A)** arterial pH and **(B)** partial pressure of carbon dioxide (pCO_2_) across seven time points (T1–T7), stratified by sentinel event (S.EVT) status: no sentinel event (blue), probable sentinel event (green), and definite sentinel event (pink). The *x*-axis groups represent each sentinel event category across timepoints. Boxes indicate the interquartile range (IQR), horizontal lines mark medians, and whiskers extend to 1.5× IQR; outliers are shown as individual points. A trimming threshold (q.trim = 0.025) was applied to reduce the influence of extreme values.

The laboratory values were then examined over time between the three different groups: known sentinel event, probable sentinel event and no sentinel event. There was a significant difference between the groups for the laboratory values over time between the 3 groups for the pH, pCO2, PTT, D-dimer and WBC (*p* < 0.05)(Figure 2).

### 3.3 Laboratory values as a marker of MRI injury

#### 3.3.1 Total gray matter score

Consistent with total injury findings, lactate, pH, pCO_2_, and base deficit showed the most consistent early associations with gray matter injury severity. Elevated ALT from T2 to T5 was associated with worsening gray matter injury (*p* < 0.05), as was elevated AST at T2 and T4 (*p* < 0.05). Lower pH at T1 and T2, lower pCO_2_ at T1 and T2, and a more negative base deficit value from T1 to T6 were each associated with increased gray matter injury scores (*p* < 0.05). Elevated lactate from T1 to T7 was also associated with increasing injury (*p* < 0.05). An increased PT–INR at T1 and T3 was significantly associated with more severe gray matter injury (*p* < 0.05). Additionally, lower total bilirubin concentrations at T1–T3, T5, and T6 were associated with more severe brain injury (*p* < 0.05)(summarized in Table 3).

**Table 3:** Associations between laboratory biomarkers and MRI weeke injury scores.

Region	Biomarker	Timepoints	Association
Gray Matter	ALT	T2, T3, T4, T5	A higher ALT was associated with higher Weeke scores
	AST	T2, T4	A higher AST was associated with higher Weeke scores
	pH	T1, T2	A lower pH was associated with higher Weeke scores
	pCO_2_	T1, T2	A lower pCO_2_ was associated with higher Weeke scores
	Base Deficit	T1, T2, T3, T4, T5, T6	A more negative deficit was associated with higher Weeke scores
	Lactate	T1, T2, T3, T4, T5, T6, T7	A higher lactate was associated with higher Weeke scores
	PT–INR	T1, T3	A higher INR was associated with higher Weeke scores
	Total Bilirubin	T1, T2, T3, T5, T6	A lower total bilirubin was associated with higher Weeke scores
	Creatinine	T2, T3	A higher creatinine was associated with higher Weeke scores
White Matter	ALT	T2, T3, T4, T5	A higher ALT was associated with higher Weeke scores
	AST	T2, T4	A higher AST was associated with higher Weeke scores
	pH	T1	A lower pH was associated with higher Weeke scores
	pCO_2_	T1	A lower pCO_2_ was associated with higher Weeke scores
	Base Deficit	T1, T2, T4	A more negative deficit was associated with higher Weeke scores
	Lactate	T1, T2, T3, T4, T5, T6	A higher lactate was associated with higher Weeke scores
	PT–INR	T1, T3	A higher INR was associated with higher Weeke scores
	D–dimer	T1, T3, T4	A higher D–dimer was associated with higher Weeke scores
	Platelet	T2, T5, T6, T7	A lower platelet count was associated with higher Weeke scores
Cerebellum	Total Bilirubin	T2	A lower total bilirubin was associated with higher Weeke scores
	Base Deficit	T7	A more negative deficit was associated with higher Weeke scores
	Lactate	T2, T3, T5, T6	A higher lactate was associated with higher Weeke scores
	D–dimer	T2, T7	A higher D–dimer was associated with higher Weeke scores
	PT–INR	T4	A higher INR was associated with higher Weeke scores
	Total Bilirubin	T1, T5	A lower total bilirubin was associated with higher Weeke scores
Total Score	ALT	T2, T3, T4, T5	A higher ALT was associated with higher Weeke scores
	AST	T2, T4	A higher AST was associated with higher Weeke scores
	pH	T1, T2	A lower pH was associated with higher Weeke scores
	pCO_2_	T1, T2	A lower pCO_2_ was associated with higher Weeke scores
	Base Deficit	T1, T2, T3, T4	A more negative deficit was associated with higher Weeke scores
	Lactate	T1, T2, T3, T4, T5, T6, T7	A higher lactate was associated with higher Weeke scores
	Total Bilirubin	T2	A lower total bilirubin was associated with higher Weeke scores
	PT–INR	T1, T3	A higher INR was associated with higher Weeke scores
	D–dimer	T1, T3, T4, T5	A higher D–dimer was associated with higher Weeke scores
	PTT	T1	A higher PTT was associated with higher Weeke scores
	Platelet	T5, T6, T7	A lower platelet count was associated with higher Weeke scores
	Cortisol	T6	A higher cortisol was associated with higher Weeke scores
	Fibrinogen	T5	A higher fibrinogen was associated with higher Weeke scores

#### 3.3.2 Total white matter score

Lactate, pH, pCO_2_, and base deficit demonstrated the most consistent early associations with white matter injury severity, with additional time-dependent relationships observed for other laboratory measures. Elevated transaminase levels correlated with higher white matter injury scores, including ALT at T2, T3, T4, and T5 (*p* < 0.05) and AST at T2 and T4 (*p* < 0.05). Additionally, lower pH (*p* < 0.05) and lower pCO_2_ (*p* < 0.05) at T1 were associated with more severe white matter injury. In addition, a more negative base deficit value at T1, T2, and T4 (*p* < 0.05) were associated with higher Weeke injury scores. Elevated lactate from T1 through T6 (*p* < 0.05) correlated with worsening white matter injury. In coagulation studies, an increased PT-INR at T1 and T3 was associated with worsening Weeke scores (*p* < 0.05). Elevated D-dimer levels at T1, T3, and T4 were associated with more severe white matter injury (*p* < 0.05). Additionally, lower platelet counts at T2, T5, T6, and T7, as well as reduced total bilirubin levels at T2, correlated with higher Weeke scores, indicating worsening white matter injury (*p* < 0.05)(summarized in Table 3).

#### 3.3.3 Total cerebellar score

Associations with cerebellar injury were less extensive, but lactate and acid–base measures remained among the most informative biomarkers. A more negative base deficit at T7 (*p* < 0.05) was associated with increased injury. Elevated lactate levels at T2, T3, T5, and T6 (*p* < 0.05) correlated with worsening Weeke scores. Additionally, elevated D-dimer levels at T2 and T7 (*p* < 0.05) were associated with greater cerebellar injury, while an increased PT-INR at T4 (*p* < 0.05) also correlated with higher cerebellar injury scores. Lower total bilirubin concentrations at T1 and T5 were similarly associated with more severe cerebellar injury (*p* < 0.05)(summarized in Table 3).

#### 3.3.4 Total brain injury score

Across evaluated laboratory markers, lactate, pH, pCO_2_, and base deficit demonstrated the strongest and most consistent associations with total brain injury severity, particularly at the earliest timepoints. Elevated ALT levels at T2, T3, T4, and T5 (*p* < 0.05) correlated with higher total brain injury scores. Elevated AST levels at T2 and T4 (*p* < 0.05) were also associated with worsening Weeke scores. Lower pH at T1 and T2 (*p* < 0.05) was significantly associated with increased brain injury severity, and decreased pCO_2_ at these same time points (*p* < 0.05) also correlated with higher injury scores. Additionally, a more negative base deficit at T1, T2, T3, and T4 (*p* < 0.05) correlated with worsening total brain injury scores. Elevated lactate levels from T1 through T7, along with lower total bilirubin concentrations at T2 (*p* < 0.05), were significantly associated with higher Weeke scores, indicating more severe overall brain injury.

Coagulation abnormalities were also observed: increased PT-INR at T1 and T3 (*p* < 0.05) correlated with worsening brain injury, while elevated D-dimer at T1, T3, T4, and T5 (*p* < 0.05) was linked to increased injury severity. Increased PTT at T1 (*p* < 0.05) was also associated with worsening Weeke scores. Elevated fibrinogen at T5 (*p* < 0.05) was significantly correlated with higher total brain injury scores, whereas a lower platelet count at T5, T6, and T7 (*p* < 0.05) was associated with worsening total brain injury scores.

Furthermore, increased cortisol at T6 (*p* < 0.05) was significantly associated with worsening Weeke scores (summarized in Table 3).

#### 3.3.5 Descriptive analysis of laboratory trends by threshold MRI score

**Figure 3:**
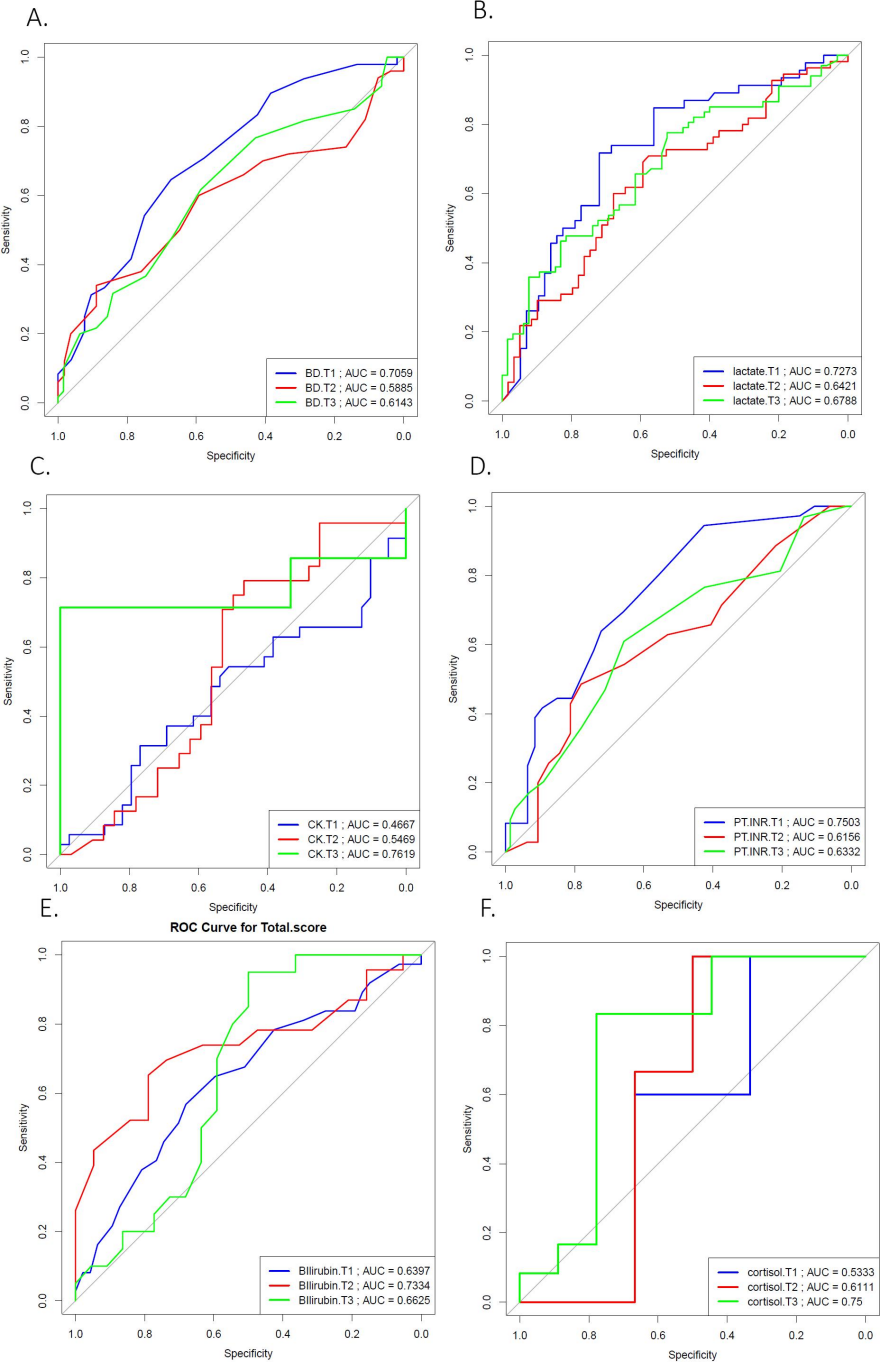
Receiver operating characteristic (ROC) curves for selected laboratory biomarkers at early time points in relation to MRI brain injury severity. ROC curves display the ability of **(A)** base deficit, **(B)** lactate, **(C)** creatine kinase (CK), **(D)** prothrombin time-international normalized ratio (PT-INR), **(E)** bilirubin, and **(F)** cortisol to discriminate between mild (MRI ≤ 2) and severe (MRI > 2) brain injury, stratified by sentinel event classification. Curves represent neonates with definite (T1, blue), probable (T2, red), and no (T3, green) sentinel events. The area under the curve (AUC) is shown for each subgroup within each panel. Diagonal gray lines indicate the reference line for random classification (AUC = 0.5).

The data was further analyzed by categorizing MRI findings into none/mild (total scores <2) and moderate/severe (total scores ≥2) for each subcategory and the total score. The area under the curve (AUC) was calculated for each biomarker from T1 to T3. The results are presented in Table 4, using a threshold of 0.7, and the corresponding AUC curves for T1-T3 are shown in Figure 3.

**Table 4:** Receiver-operating-characteristic (ROC) performance of biomarkers for MRI-defined brain injury.

Biomarker	Timepoint	AUC	Sensitivity	Specificity	Positive predictive value	Negative predictive value
White Matter						
pCO_2_	T1	0.73	0.54	0.85	0.58	0.82
Base Deficit	T1	0.74	0.78	0.63	0.44	0.88
Base Deficit	T2	0.74	0.74	0.69	0.45	0.88
Lactate	T1	0.8	0.92	0.68	0.48	0.96
Lactate	T3	0.72	0.94	0.43	0.38	0.95
PT-INR	T1	0.73	0.89	0.47	0.32	0.94
Gray Matter						
ALT	T2	0.76	0.69	0.73	0.58	0.81
AST	T2	0.77	0.56	0.9	0.75	0.79
pCO_2_	T1	0.75	0.6562	0.75	0.55	0.82
Base Deficit	T1	0.74	0.6562	0.7353	0.54	0.82
Lactate	T1	0.73	0.7667	0.6438	0.47	0.87
PT-INR	T1	0.75	0.7273	0.6721	0.44	0.87
Creatinine	T2	0.71	0.13	0.53	0.09	0.65
Creatinine	T3	0.72	0.24	0.3	0.14	0.47
Cerebellum						
ALT	T3	0.98	1	0.98	0.5	1
AST	T1	0.74	1	0.73	0.04	1
pCO_2_	T2	0.73	0.5	0.97	0.25	1
pCO_2_	T3	0.7	1	0.49	0.03	1
Lactate	T2	0.92	1	0.92	0.18	1
PTT	T1	0.86	1	0.76	0.11	1
D-Dimer	T1	0.86	1	0.71	0.1	1
D-Dimer	T2	0.84	1	0.70	0.09	1
D-Dimer	T3	0.85	1	0.78	0.06	1
Platelet	T1	0.8	1	0.75	0.09	1
Platelet	T2	0.76	1	0.72	0.09	1
Platelet	T3	0.8	1	0.71	0.05	1
Total Score						
Base Deficit	T1	0.7	0.65	0.67	0.65	0.67
Lactate	T1	0.72	0.72	0.72	0.67	0.76
PT-INR	T1	0.75	0.94	0.43	0.56	0.91

#### 3.3.6 Use of multiple biomarkers to improve predictive performance

Model selection strategies for multiple linear regression were used to identify key predictors of the Total Brain Injury Scores across timepoints T1–T3. Using the imputed dataset (larger sample size), all possible subsets of predictors were evaluated, and the optimal model was selected based on Schwarz's Bayesian Information Criterion (BIC), which penalizes model complexity to minimize overfitting. This selection was subsequently confirmed using the observed dataset (smaller sample size due to missing predictor values).

The BIC-selected model included pH at T1, pCO2 at T1, and lactate at T3, achieving an adjusted R^2^ of 46.8% on the observed dataset and 34.1% on the imputed dataset. Coefficient estimates and model statistics are presented in Table 5. This level of explained variance reflects predictive performance and should be interpreted as supporting early risk stratification rather than definitive clinical prediction.

**Table 5:** Multiple linear regression models predicting outcome based on laboratory values at timepoints T1–T3.

Predictor	Estimate (*β*)	Std. Error	*t* value	*p*-value
Model 1: BIC-selected (pH.T1, pCO2.T1, lactate.T3)				
Intercept	109.96	36.04	3.05	0.003
pH.T1	−13.91	4.89	−2.84	0.006
pCO2.T1	−0.206	0.036	−5.76	< 0.001
lactate.T3	1.081	0.182	5.93	< 0.001
Model statistics	Adj. R^2^ = 0.468	Residual SE = 5.55 (df = 91)		
Model 2: Conservative (pCO2.T1, lactate.T3)				
Intercept	7.53	1.55	4.85	< 0.001
pCO2.T1	−0.163	0.034	−4.86	< 0.001
lactate.T3	1.264	0.175	7.21	< 0.001
Model statistics	Adj. R^2^ = 0.427	Residual SE = 5.74 (df = 93)		

SE, standard error; Adj. R^2^, adjusted R-squared; BIC, Bayesian Information Criterion; SE, standard error.

To assess the stability of model selection, five-fold cross-validation was performed on the imputed dataset (∼80% of data per fold). Variables most frequently selected included pCO2 at T1 (5/5 folds) and lactate at T3 (5/5 folds), followed by glucose at T1 (3/5 folds), pH at T1 (2/5 folds), BD at T2 (2/5 folds), BD at T3 (1/5 folds), and fibrinogen at T3 (1/5 folds). A conservative two-predictor model including pCO2 at T1 and lactate at T3 was therefore examined, achieving an adjusted R^2^ of 42.7% on the observed dataset (29.6% on the imputed dataset) (Table 5).

Single-predictor models demonstrated weaker predictive performance. Across T1–T3 timepoints, lactate at T3 was the strongest individual predictor (adjusted R^2^ = 25.1%). Within T1 alone, the best single predictors were BD at T1 (21.1%) and lactate at T1 (20.4%). Multivariable T1-only models provided incremental improvements, with adjusted R^2^ values of 27.1% for pH at T1 + pCO2 at T1, 29.5% for pCO2 at T1 + lactate at T1, and 31.6% for pCO2 at T1 + lactate at T1 + pH at T1 (Table 6). Overall, models incorporating multiple predictors across timepoints (T1–T3) improved the absolute adjusted R^2^ score by approximately 15–20 percentage points, which translates into the relative predictive performance boost of 60%–80% (approximately 0.15/0.25–0.2/0.25) compared to the best single-predictor models, despite the adverse effects of missing data.

**Table 6:** Summary of predictive performance (adjusted R^2^) for single-predictor and T1-only multiple linear regression models.

Model Type	Predictors included	Adjusted R^2^ (%)
Single predictors (T1–T3)	lactate.T3	25.1
Single predictors (T1 only)	BD.T1	21.1
	lactate.T1	20.4
T1-only multivariable models	pH.T1 + pCO2.T1 (BIC-selected)	27.1
	pCO2.T1 + lactate.T1	29.5
	pCO2.T1 + lactate.T1 + pH.T1	31.6

Adj. R^2^, adjusted R-squared; BIC, Bayesian Information Criterion; BD, base deficit. Models are based on observed datasets (n reduced due to missing values).

## 4 Discussion

This single-center retrospective cohort study examined temporal trends in laboratory biomarkers among neonates with hypoxic-ischemic encephalopathy (HIE) treated with therapeutic hypothermia (TH). Laboratory values were collected at seven predefined time points from birth through beyond 96 h of life, encompassing the entire course of cooling and rewarming. This longitudinal approach enabled a comprehensive evaluation of how bedside laboratory values, including liver enzymes, acid-base status, renal function, cardiac injury indicators, and coagulation parameters, changed over time, providing clinicians with an expected temporal profile of laboratory trends following HIE. Most abnormalities were observed within the first 24 h of life, with gradual normalization thereafter. These data provide an expected clinical trajectory for laboratory values in neonates with HIE undergoing therapeutic hypothermia. Subsequently, the relationship between routine bedside laboratory values and MRI-defined brain injury was investigated to assess whether these laboratory results could serve as surrogate indicators of neurologic injury. MRI brain injury scores, assessed using the Weeke classification, were analyzed for associations with laboratory markers reflective of multisystem injury and physiologic disturbances, and their correlation with the severity of brain injury. Among evaluated laboratory markers, lactate, pH, pCO_2_, and base deficit demonstrated the most consistent early associations with MRI-defined brain injury, while transaminases and coagulation parameters showed additional time-dependent relationships.

Similar temporal trajectories in laboratory biomarkers have been reported in prior studies of neonates with HIE; however, these investigations typically included less frequent sampling, limiting the precision of temporal characterization. By incorporating more frequent and extended intervals, our study provides improved temporal resolution and refines previously described laboratory trends before and after rewarming (3, 16, 17). In our study, hepatic enzymes demonstrated a distinct injury-recovery trajectory. Alanine aminotransferase (ALT) levels peaked early, most prominently at T2 (6–12 h after birth), and remained elevated over several days, while aspartate aminotransferase (AST) peaked at T2 and declined steadily between 48 and 96 h. Previous studies have reported elevations in ALT and AST within the first 24 h and a peak between 24 and 72 h in neonates with birth asphyxia who did not undergo therapeutic hypothermia (17, 18). Our findings differ in that we included three sampling points within the first 24 h, allowing for a more granular understanding of early hepatic enzyme dynamics. This detailed temporal profiling provides insights into the timing and evolution of liver injury in neonates with HIE receiving neuroprotective treatment.

Metabolic indicators such as arterial pH, pCO_2_, base deficit, and lactate showed rapid improvement over time, with the most severe derangements observed at T1 and resolution generally occurring by T4. Coagulation markers revealed a pattern of early coagulopathy, with prolonged PTT and elevated PT-INR at baseline that improved over time, while fibrinogen levels rose steadily from T3 onward. Cardiac injury markers showed transient elevations: CK peaked at T2 and declined thereafter. Cortisol levels varied widely, with a general decline over time, and creatinine levels reflected improving renal function across later timepoints. Collectively, these patterns highlight a systemic response to hypoxic-ischemic injury and recovery and demonstrate the value of serial lab monitoring in assessing organ function trajectories in HIE and underscores the importance of early and serial monitoring of specific laboratory biomarkers in neonates with HIE undergoing therapeutic hypothermia. The observed temporal patterns may help clinicians anticipate evolving organ dysfunction, adjust supportive therapies proactively, and identify infants at higher risk for adverse outcomes.

The typical postnatal increase in bilirubin observed in neonates not undergoing therapeutic hypothermia is primarily attributed to enhanced hemolysis of fetal erythrocytes, immature hepatic conjugation capacity due to reduced UDP-glucuronosyltransferase (UGT1A1) activity, and increased enterohepatic circulation (19). In contrast to the expected physiologic rise in bilirubin among term neonates during the first few days of life, our cohort demonstrated comparatively lower peak bilirubin levels during therapeutic hypothermia (20). One potential explanation for this finding is that neonates with moderate to severe HIE have been shown to exhibit reduced heme oxygenase activity, the rate-limiting enzyme in the production of bilirubin (20, 21). In our cohort, lower total bilirubin concentrations at 12, 24, 72, and 96 h were significantly associated with more severe brain injury, as quantified by the Weeke MRI scores. Although speculative, this inverse relationship raises the possibility that bilirubin's antioxidant properties may confer neuroprotection in the setting of HIE (22). Alternatively, lower bilirubin levels in infants with more severe hypoxic-ischemic injury may reflect increased oxidative stress related consumption of bilirubin, a hypothesis that remains speculative and warrants further investigation (23). When the cohort was stratified into three groups—definite sentinel event, probable sentinel event, and no sentinel event, distinct differences in laboratory trends over time were observed, particularly for pH, partial pressure of carbon dioxide (pCO_2_), partial thromboplastin time (PTT), D-dimer, and white blood cell (WBC) count. The no sentinel event group exhibited the lowest pH and highest pCO_2_ values, suggesting more profound metabolic and respiratory derangements. In contrast, the definite and probable sentinel event groups demonstrated higher pH values and comparatively lower pCO_2_ levels. Prior studies have recognized that sentinel events influence biochemical findings in HIE, but have not characterized longitudinal laboratory changes. This study adds to this work by showing that temporal laboratory patterns differ across sentinel event categories (1, 3, 24). These findings are noteworthy, as they may provide insights into the timing and nature of the hypoxic-ischemic insult. Additionally, our study's stratification approach offers insights into metabolic and coagulation profiles, contributing to the growing body of literature on the importance of contextualizing laboratory values within the clinical presentation of HIE (3, 4, 17, 25–27). However, validation in a larger, prospective cohort is necessary to confirm these associations and establish their clinical utility in injury timing.

Our study also aimed to evaluate the association between routine bedside laboratory values and MRI-defined brain injury, exploring the potential of these systemic parameters to serve as early biomarkers of neurologic injury. Furthermore, aligning laboratory trajectories with MRI findings offers a framework to refine prognostic discussions with families and tailor follow-up strategies based on individual risk profiles. Our findings demonstrated that pCO_2_, lactate, ALT, AST, pH, PT-INR, PTT and D-dimer, were significantly associated with brain injury patterns as assessed using the Weeke scoring system (9). Consistent with our findings, several previous studies have demonstrated that lower pCO_2_ levels are associated with more severe MRI-defined brain injury in neonates with HIE (28–31). Elevated lactate concentrations were associated with short-term MRI-defined brain injury involving the gray matter, white matter, and cerebellum in our study. Prior research has shown that lactate levels are reliable predictors of long-term adverse neurological outcomes in neonates with HIE undergoing therapeutic hypothermia, as assessed at one to two years of age using the Bayley Scales of Infant and Toddler Development (32, 33). The observed abnormalities in coagulation markers in our study, including elevated D-dimer and prolonged PTT, align with findings from Sweetman et al., who reported that prolonged PT and PTT on days 1, 2, and 3 after birth in neonates undergoing TH correlated with MRI injury patterns and adverse outcomes in cooled neonates (34). Consistent with findings from the Sweetman study, we observed that a prolonged prothrombin time (PT) at 48 h and partial thromboplastin time (PTT) at 24 h were associated with MRI-defined brain injury. Additionally, prolonged PT and PTT at 96 h were significantly correlated with higher Weeke MRI scores, indicating more severe injury. In conclusion, our findings support the potential role of laboratory biomarkers as early indicators of neurological injury in neonates with HIE. These markers may ultimately facilitate more personalized therapeutic approaches and enhance risk stratification to improve clinical outcomes.

In this study, we employed predictive modeling to integrate clinical laboratory values obtained during three early postnatal time intervals (0–6 h, 7–12 h, and 13–24 h) with the aim of enhancing prognostic accuracy for neonatal brain injury. Our analysis demonstrated that lactate, partial pressure of carbon dioxide (pCO_2_), and pH measured within the first 0–6 h of life had the highest predictive value for total brain injury, as quantified by the Weeke Score. This combined modeling approach provides a novel framework for the development of predictive calculators in future studies, enabling early identification of neonates at greatest risk for brain injury within the critical first hours of life and potentially informing timely, targeted clinical interventions. When performing variable selection, we did not impose heredity constraints on the inclusion of biomarkers from the first three timepoints or interactions (e.g., if a biomarker at a later timepoint is included, then this necessitates inclusion of this biomarker at earlier timepoints); however, such extensions are possible for quantifying biomarker value for early detection of the injury (35). Within the first 6–12 h of life, lactate, pH, pCO_2_, and base deficit demonstrated the strongest and most consistent associations with total MRI injury severity. Early integration of these routinely available laboratory values may support risk stratification during the initial postnatal period while clinical evolution is pending.

Our study has several limitations that warrant consideration. First, the single-center design may limit the generalizability of our findings, as clinical practices, patient populations, and therapeutic hypothermia management protocols can vary across institutions. The degree to which such inter-institutional variability impacts laboratory trends or MRI outcomes in neonates with HIE remains uncertain. Second, the timing of laboratory sample collection was not uniform across subjects; instead, samples were grouped into predefined postnatal time intervals, with some time points containing missing values. This variability may have reduced the temporal resolution of analyses and introduced potential bias. Furthermore, it is important to consider that some laboratory derangements may reflect therapy-related effects rather than primary injury. For example, platelet dysfunction observed during hypothermia may be iatrogenic and reversible, rather than a direct consequence of the initial hypoxic insult (36). Because half of the study population was outborn, laboratory values were more frequently missing at the 0–6-hour timepoint, with fewer missing values at later timepoints. Although a protocol specified laboratory sampling timepoints, bedside clinicians could reduce the number of samples based on the neonate's clinical course, resulting in missing laboratory measurements at later timepoints. Due to the missing data, we statistically evaluated whether the pattern of missingness of the biomarkers selected as best predictors in the multivariable predictive models was informative, by comparing the distributions of the response (MRI) and the selected predictors in the “complete data” group and the “partially observed” group. Specifically, using the Kolmogorov–Smirnov test (*p* > 0.36) and the Kruskal–Wallis test (*p* > 0.11), we found no evidence of a “distribution shift” in the response (MRI); the results for selected predictors were similar. As the distributions of these variables did not differ statistically across the two groups, this investigation provides evidence against the hypothesis of informative data missingness (e.g., where missing predictors could be associated with more severe cases) and suggests that subjects with partially missing biomarkers were not clinically distinct from those with complete data. The fact that the “complete data” analyses had stronger association metrics (R^2^) than the “imputed data” analyses further reaffirms the conservatism of our analytic approach. However, this also suggests a limitation of our dataset that a very informative clinical predictor may not have been identified due to the missingness, either due to high proportion of missing values or due to the “signal attenuation” after the imputation. Not all MRIs were obtained within the 3–6-day window, which may have introduced variability in injury assessment. In addition, not all subjects experienced a clearly defined sentinel event, introducing uncertainty in the timing of injury onset that may have influenced measured laboratory concentrations. Because sentinel event classification was performed retrospectively and relied on available clinical documentation, some degree of misclassification is possible, particularly among outborn infants with incomplete perinatal records. Finally, the retrospective classification of sentinel events relied on clinical documentation, which is inherently subject to misclassification, particularly given that approximately 58% of the cohort was outborn, limiting access to complete perinatal records. This limitation may have affected the accuracy of group stratification and subsequent interpretation of laboratory trends by sentinel event status.

## 5 Conclusion

This study provides a characterization of laboratory value trajectories in neonates with HIE undergoing therapeutic hypothermia, highlighting their temporal evolution and potential prognostic significance. Key laboratory parameters reflecting hepatic, metabolic, coagulation, and inflammatory responses exhibited distinct patterns during the cooling and rewarming phases. Notably, early abnormalities in ALT, AST, lactate, base deficit, and coagulation measures were significantly associated with MRI-defined brain injury severity across multiple brain regions. Stratification by sentinel event status further revealed group-specific laboratory trends that may reflect differences in the timing and nature of the hypoxic insult.

These findings underscore the clinical utility of serial laboratory monitoring not only for guiding supportive care but also as potential early indicators of neurologic injury. Integrating these values into predictive frameworks may improve risk stratification, enhance prognostic counseling, and inform targeted neuroprotective strategies. Furthermore, we hope that combining specific laboratory values into composite models will enhance diagnostic accuracy and allow for earlier identification of neonates at highest risk for brain injury. Future multicenter, prospective studies with standardized data collection and long-term neurodevelopmental follow-up are warranted to validate these associations and refine laboratory-based approaches to personalized care in neonates with HIE.

## Data Availability

The raw data supporting the conclusions of this article will be made available by the authors, without undue reservation.
