# Supplementary material for: Lab values in neonates with hypoxic ischemic encephalopathy over time during and after therapeutic hypothermia
Source: Front Pediatr. 2026 Mar 12;14:1743749. doi: 10.3389/fped.2026.1743749 (PMC13017858; doi:10.3389/fped.2026.1743749)
Supplement: Supplementary file 1 [file Table1.docx]

**Supplementary Table 1.** Comparisons of systemic biomarker concentrations between T1 and subsequent timepoints (T2–T7).

| Biomarker | Comparison | p-value |
| --- | --- | --- |
| ALT | T1 vs T2 | 0.0003 |
| ALT | T1 vs T3 | 0.0048 |
| ALT | T1 vs T4 | <0.0001 |
| ALT | T1 vs T5 | 0.0157 |
| ALT | T1 vs T6 | 0.4428 |
| ALT | T1 vs T7 | 0.2743 |
| AST | T1 vs T2 | <0.0001 |
| AST | T1 vs T3 | 0.0027 |
| AST | T1 vs T4 | 0.2635 |
| AST | T1 vs T5 | 0.0245 |
| AST | T1 vs T6 | 0.0007 |
| AST | T1 vs T7 | <0.0001 |
| Bilirubin | T1 vs T2 | 0.0623 |
| Bilirubin | T1 vs T3 | <0.0001 |
| Bilirubin | T1 vs T4 | <0.0001 |
| Bilirubin | T1 vs T5 | <0.0001 |
| Bilirubin | T1 vs T6 | <0.0001 |
| Bilirubin | T1 vs T7 | <0.0001 |
| pH | T1 vs T2 | 0.0016 |
| pH | T1 vs T3 | <0.0001 |
| pH | T1 vs T4 | <0.0001 |
| pH | T1 vs T5 | <0.0001 |
| pH | T1 vs T6 | <0.0001 |
| pH | T1 vs T7 | <0.0001 |
| pCO2 | T1 vs T2 | 0.2179 |
| pCO2 | T1 vs T3 | 0.0090 |
| pCO2 | T1 vs T4 | <0.0001 |
| pCO2 | T1 vs T5 | <0.0001 |
| pCO2 | T1 vs T6 | <0.0001 |
| pCO2 | T1 vs T7 | <0.0001 |
| BD | T1 vs T2 | <0.0001 |
| BD | T1 vs T3 | <0.0001 |
| BD | T1 vs T4 | <0.0001 |
| BD | T1 vs T5 | <0.0001 |
| BD | T1 vs T6 | <0.0001 |
| BD | T1 vs T7 | <0.0001 |
| Lactate | T1 vs T2 | <0.0001 |
| Lactate | T1 vs T3 | <0.0001 |
| Lactate | T1 vs T4 | <0.0001 |
| Lactate | T1 vs T5 | <0.0001 |
| Lactate | T1 vs T6 | <0.0001 |
| Lactate | T1 vs T7 | <0.0001 |
| PTT | T1 vs T2 | 0.5588 |
| PTT | T1 vs T3 | <0.0001 |
| PTT | T1 vs T4 | <0.0001 |
| PTT | T1 vs T5 | 0.0044 |
| PTT | T1 vs T6 | 0.0082 |
| PTT | T1 vs T7 | 0.0003 |
| d-dimer | T1 vs T2 | 0.7597 |
| d-dimer | T1 vs T3 | 0.0007 |
| d-dimer | T1 vs T4 | <0.0001 |
| d-dimer | T1 vs T5 | <0.0001 |
| d-dimer | T1 vs T6 | <0.0001 |
| d-dimer | T1 vs T7 | <0.0001 |
| PT-INR | T1 vs T2 | 0.5220 |
| PT-INR | T1 vs T3 | <0.0001 |
| PT-INR | T1 vs T4 | <0.0001 |
| PT-INR | T1 vs T5 | <0.0001 |
| PT-INR | T1 vs T6 | <0.0001 |
| PT-INR | T1 vs T7 | <0.0001 |
| Fibrinogen | T1 vs T2 | 0.9886 |
| Fibrinogen | T1 vs T3 | 0.0004 |
| Fibrinogen | T1 vs T4 | <0.0001 |
| Fibrinogen | T1 vs T5 | <0.0001 |
| Fibrinogen | T1 vs T6 | <0.0001 |
| Fibrinogen | T1 vs T7 | <0.0001 |
| WBC | T1 vs T2 | 0.0048 |
| WBC | T1 vs T3 | 0.0076 |
| WBC | T1 vs T4 | <0.0001 |
| WBC | T1 vs T5 | <0.0001 |
| WBC | T1 vs T6 | <0.0001 |
| WBC | T1 vs T7 | <0.0001 |
| Platelet | T1 vs T2 | 0.0359 |
| Platelet | T1 vs T3 | 0.0005 |
| Platelet | T1 vs T4 | <0.0001 |
| Platelet | T1 vs T5 | <0.0001 |
| Platelet | T1 vs T6 | <0.0001 |
| Platelet | T1 vs T7 | <0.0001 |
| CK | T1 vs T2 | 0.0002 |
| CK | T1 vs T3 | 0.2212 |
| CK | T1 vs T4 | 0.3647 |
| CK | T1 vs T5 | 0.6708 |
| CK | T1 vs T6 | 0.3335 |
| CK | T1 vs T7 | 0.3096 |
| Glucose | T1 vs T2 | 0.2593 |
| Glucose | T1 vs T3 | 0.0120 |
| Glucose | T1 vs T4 | <0.0001 |
| Glucose | T1 vs T5 | 0.0004 |
| Glucose | T1 vs T6 | <0.0001 |
| Glucose | T1 vs T7 | <0.0001 |
| Cortisol | T1 vs T2 | 0.3835 |
| Cortisol | T1 vs T3 | 0.9159 |
| Cortisol | T1 vs T4 | 0.0944 |
| Cortisol | T1 vs T5 | 0.1307 |
| Cortisol | T1 vs T6 | 0.0433 |
| Cortisol | T1 vs T7 | 0.3619 |
| Creatinine | T1 vs T2 | 0.3353 |
| Creatinine | T1 vs T3 | 0.0282 |
| Creatinine | T1 vs T4 | <0.0001 |
| Creatinine | T1 vs T5 | <0.0001 |
| Creatinine | T1 vs T6 | <0.0001 |
| Creatinine | T1 vs T7 | <0.0001 |

Caption: This table presents p-values for within-subject changes in biomarker levels across the therapeutic hypothermia and rewarming period, with T1 serving as baseline.
