# Supplementary material for: Lab values in neonates with hypoxic ischemic encephalopathy over time during and after therapeutic hypothermia
Source: Front Pediatr. 2026 Mar 12;14:1743749. doi: 10.3389/fped.2026.1743749 (PMC13017858; doi:10.3389/fped.2026.1743749)
Supplement: Supplementary file 2 [file Table2.docx]

**Supplementary Table 2.** Associations between systemic biomarkers and Grey Matter MRI injury scores across timepoints.

| Biomarker | Timepoint | p-value |
| --- | --- | --- |
| ALT | T1 | 0.4146 |
| ALT | T2 | 0.0004 |
| ALT | T3 | 0.0145 |
| ALT | T4 | 0.0018 |
| ALT | T5 | 0.0347 |
| ALT | T6 | 0.5903 |
| ALT | T7 | 0.3073 |
| AST | T1 | 0.1841 |
| AST | T2 | 0.0007 |
| AST | T3 | 0.4969 |
| AST | T4 | 0.0044 |
| AST | T5 | 0.3004 |
| AST | T6 | 0.8353 |
| AST | T7 | 0.1924 |
| Bilirubin | T1 | 0.0118 |
| Bilirubin | T2 | 0.0103 |
| Bilirubin | T3 | 0.0554 |
| Bilirubin | T4 | 0.4853 |
| Bilirubin | T5 | 0.0218 |
| Bilirubin | T6 | 0.0801 |
| Bilirubin | T7 | 0.6063 |
| pH | T1 | 0.0259 |
| pH | T2 | 0.0703 |
| pH | T3 | 0.1316 |
| pH | T4 | 0.1331 |
| pH | T5 | 0.8941 |
| pH | T6 | 0.9504 |
| pH | T7 | 0.6113 |
| pCO2 | T1 | 0.0002 |
| pCO2 | T2 | 0.0243 |
| pCO2 | T3 | 0.1188 |
| pCO2 | T4 | 0.2316 |
| pCO2 | T5 | 0.2622 |
| pCO2 | T6 | 0.6536 |
| pCO2 | T7 | 0.8939 |
| BD | T1 | <0.0001 |
| BD | T2 | 0.0002 |
| BD | T3 | 0.0017 |
| BD | T4 | 0.0006 |
| BD | T5 | 0.5034 |
| BD | T6 | 0.6194 |
| Bd | T7 | 0.4820 |
| Lactate | T1 | <0.0001 |
| Lactate | T2 | <0.0001 |
| Lactate | T3 | <0.0001 |
| Lactate | T4 | <0.0001 |
| Lactate | T5 | <0.0001 |
| Lactate | T6 | 0.0007 |
| Lactate | T7 | 0.0369 |
| PTT | T1 | 0.0013 |
| PTT | T2 | 0.5727 |
| PTT | T3 | 0.3811 |
| PTT | T4 | 0.1470 |
| PTT | T5 | 0.1094 |
| PTT | T6 | 0.0810 |
| PTT | T7 | 0.7188 |
| d.dimer | T1 | 0.0530 |
| d.dimer | T2 | 0.8191 |
| d.dimer | T3 | 0.0067 |
| d.dimer | T4 | 0.0247 |
| d.dimer | T5 | 0.0800 |
| d.dimer | T6 | 0.6578 |
| d.dimer | T7 | 0.7932 |
| PT-INR | T1 | 0.0003 |
| PT-INR | T2 | 0.3106 |
| PT-INR | T3 | 0.0070 |
| PT-INR | T4 | 0.7366 |
| PT-INR | T5 | 0.6133 |
| PT-INR | T6 | 0.5022 |
| PT-INR | T7 | 0.6314 |
| Fibrinogen | T1 | 0.2221 |
| Fibrinogen | T2 | 0.2546 |
| Fibrinogen | T3 | 0.4105 |
| Fibrinogen | T4 | 0.8277 |
| Fibrinogen | T5 | 0.0396 |
| Fibrinogen | T6 | 0.5425 |
| Fibrinogen | T7 | 0.4076 |
| WBC | T1 | 0.0516 |
| WBC | T2 | 0.6579 |
| WBC | T3 | 0.1225 |
| WBC | T4 | 0.8088 |
| WBC | T5 | 0.5675 |
| WBC | T6 | 0.8351 |
| WBC | T7 | 0.5181 |
| Platelet | T1 | 0.6772 |
| Platelet | T2 | 0.1151 |
| Platelet | T3 | 0.5137 |
| Platelet | T4 | 0.5976 |
| Platelet | T5 | 0.0476 |
| Platelet | T6 | 0.0174 |
| Platelet | T7 | 0.0364 |
| CK | T1 | 0.4026 |
| CK | T2 | 0.9326 |
| CK | T3 | 0.3999 |
| CK | T4 | 0.1637 |
| CK | T5 | 0.0089 |
| CK | T6 | 0.5326 |
| CK | T7 | 0.2320 |
| Glucose | T1 | 0.0240 |
| Glucose | T2 | 0.0557 |
| Glucose | T3 | 0.3090 |
| Glucose | T4 | 0.0728 |
| Glucose | T5 | 0.0004 |
| Glucose | T6 | 0.0082 |
| Glucose | T7 | 0.0014 |
| Cortisol | T1 | 0.5817 |
| Cortisol | T2 | 0.6724 |
| Cortisol | T3 | 0.4440 |
| Cortisol | T4 | 0.1958 |
| Cortisol | T5 | 0.8800 |
| Cortisol | T6 | 0.1869 |
| Cortisol | T7 | 0.4978 |
| Creatinine | T1 | 0.9190 |
| Creatinine | T2 | 0.5537 |
| Creatinine | T3 | 0.6841 |
| Creatinine | T4 | 0.7014 |
| Creatinine | T5 | 0.6998 |
| Creatinine | T6 | 0.4563 |
| Creatinine | T7 | 0.5880 |

Caption: P-values for the associations between individual biomarker concentrations at each timepoint and Grey Matter injury severity.
