# Supplementary material for: Lab values in neonates with hypoxic ischemic encephalopathy over time during and after therapeutic hypothermia
Source: Front Pediatr. 2026 Mar 12;14:1743749. doi: 10.3389/fped.2026.1743749 (PMC13017858; doi:10.3389/fped.2026.1743749)
Supplement: Supplementary file 3 [file Table3.docx]

**Supplementary Table 3.** Associations between systemic biomarkers and White Matter MRI injury scores across timepoints.

| Biomarker | Timepoint | p-value |
| --- | --- | --- |
| ALT | T1 | 0.6713 |
| ALT | T2 | 0.0027 |
| ALT | T3 | 0.0334 |
| ALT | T4 | 0.0045 |
| ALT | T5 | 0.0432 |
| ALT | T6 | 0.3870 |
| ALT | T7 | 0.9122 |
| AST | T1 | 0.7290 |
| AST | T2 | 0.0014 |
| AST | T3 | 0.9606 |
| AST | T4 | 0.0367 |
| AST | T5 | 0.4111 |
| AST | T6 | 0.8764 |
| AST | T7 | 0.9176 |
| Bilirubin | T1 | 0.2122 |
| Bilirubin | T2 | 0.0025 |
| Bilirubin | T3 | 0.0702 |
| Bilirubin | T4 | 0.2391 |
| Bilirubin | T5 | 0.0374 |
| Bilirubin | T6 | 0.0916 |
| Bilirubin | T7 | 0.8044 |
| pH | T1 | 0.0189 |
| pH | T2 | 0.0722 |
| pH | T3 | 0.2086 |
| pH | T4 | 0.5988 |
| pH | T5 | 0.7297 |
| pH | T6 | 0.9855 |
| pH | T7 | 0.4965 |
| pCO2 | T1 | 0.0032 |
| pCO2 | T2 | 0.0907 |
| pCO2 | T3 | 0.8760 |
| pCO2 | T4 | 0.6000 |
| pCO2 | T5 | 0.3118 |
| pCO2 | T6 | 0.4525 |
| pCO2 | T7 | 0.6948 |
| BD | T1 | <0.0001 |
| BD | T2 | <0.0001 |
| BD | T3 | 0.2430 |
| BD | T4 | 0.0337 |
| BD | T5 | 0.7587 |
| BD | T6 | 0.4773 |
| BD | T7 | 0.4808 |
| lactate | T1 | <0.0001 |
| lactate | T2 | 0.0002 |
| lactate | T3 | <0.0001 |
| lactate | T4 | 0.0040 |
| lactate | T5 | 0.0049 |
| lactate | T6 | 0.0004 |
| lactate | T7 | 0.0245 |
| PTT | T1 | 0.1203 |
| PTT | T2 | 0.3618 |
| PTT | T3 | 0.1794 |
| PTT | T4 | 0.0594 |
| PTT | T5 | 0.0775 |
| PTT | T6 | 0.3413 |
| PTT | T7 | 0.7060 |
| d.dimer | T1 | 0.0118 |
| d.dimer | T2 | 0.3058 |
| d.dimer | T3 | <0.0001 |
| d.dimer | T4 | 0.0157 |
| d.dimer | T5 | 0.0830 |
| d.dimer | T6 | 0.9266 |
| d.dimer | T7 | 0.2868 |
| PT-INR | T1 | 0.0015 |
| PT-INR | T2 | 0.3843 |
| PT-INR | T3 | 0.0004 |
| PT-INR | T4 | 0.8302 |
| PT-INR | T5 | 0.5462 |
| PT-INR | T6 | 0.5529 |
| PT-INR | T7 | 0.6704 |
| Fibrinogen | T1 | 0.1713 |
| Fibrinogen | T2 | 0.4053 |
| Fibrinogen | T3 | 0.5743 |
| Fibrinogen | T4 | 0.2173 |
| Fibrinogen | T5 | 0.0024 |
| Fibrinogen | T6 | 0.1605 |
| Fibrinogen | T7 | 0.1742 |
| WBC | T1 | 0.2288 |
| WBC | T2 | 0.9631 |
| WBC | T3 | 0.0675 |
| WBC | T4 | 0.6885 |
| WBC | T5 | 0.4838 |
| WBC | T6 | 0.6526 |
| WBC | T7 | 0.7145 |
| Platelet | T1 | 0.8350 |
| Platelet | T2 | 0.0174 |
| Platelet | T3 | 0.1640 |
| Platelet | T4 | 0.2574 |
| Platelet | T5 | 0.0143 |
| Platelet | T6 | 0.0081 |
| Platelet | T7 | 0.0011 |
| CK | T1 | 0.4770 |
| CK | T2 | 0.8580 |
| CK | T3 | 0.4151 |
| CK | T4 | 0.8248 |
| CK | T5 | 0.8167 |
| CK | T6 | 0.5326 |
| CK | T7 | 0.1313 |
| Glucose | T1 | 0.0180 |
| Glucose | T2 | 0.0542 |
| Glucose | T3 | 0.9152 |
| Glucose | T4 | 0.0212 |
| Glucose | T5 | 0.0002 |
| Glucose | T6 | 0.0002 |
| Glucose | T7 | 0.0587 |
| Cortisol | T1 | 0.6641 |
| Cortisol | T2 | 0.8352 |
| Cortisol | T3 | 0.7875 |
| Cortisol | T4 | 0.4696 |
| Cortisol | T5 | 0.8688 |
| Cortisol | T6 | 0.0800 |
| Cortisol | T7 | 0.7314 |
| Creatinine | T1 | 0.7877 |
| Creatinine | T2 | 0.9480 |
| Creatinine | T3 | 0.9034 |
| Creatinine | T4 | 0.9936 |
| Creatinine | T5 | 0.8426 |
| Creatinine | T6 | 0.9408 |
| Creatinine | T7 | 0.9912 |

Caption: P-values for the associations between biomarker concentrations at each timepoint and White Matter injury severity.
