# Supplementary material for: Lab values in neonates with hypoxic ischemic encephalopathy over time during and after therapeutic hypothermia
Source: Front Pediatr. 2026 Mar 12;14:1743749. doi: 10.3389/fped.2026.1743749 (PMC13017858; doi:10.3389/fped.2026.1743749)
Supplement: Supplementary file 4 [file Table4.docx]

**Supplementary Table 4.** Associations between systemic biomarkers and Cerebellar MRI injury scores across timepoints.

| Biomarker | Timepoint | p-value |
| --- | --- | --- |
| ALT | T1 | 0.5181 |
| ALT | T2 | 0.1333 |
| ALT | T3 | 0.6089 |
| ALT | T4 | 0.9421 |
| ALT | T5 | 0.5660 |
| ALT | T6 | 0.2956 |
| ALT | T7 | 0.4816 |
| AST | T1 | 0.3701 |
| AST | T2 | 0.7179 |
| AST | T3 | 0.6059 |
| AST | T4 | 0.9814 |
| AST | T5 | 0.6015 |
| AST | T6 | 0.8348 |
| AST | T7 | 0.5163 |
| Bilirubin | T1 | 0.0394 |
| Bilirubin | T2 | 0.2017 |
| Bilirubin | T3 | 0.7550 |
| Bilirubin | T4 | 0.4972 |
| Bilirubin | T5 | 0.0128 |
| Bilirubin | T6 | 0.9347 |
| Bilirubin | T7 | 0.0047 |
| pH | T1 | 0.3223 |
| pH | T2 | 0.9674 |
| pH | T3 | 0.0801 |
| pH | T4 | 0.6412 |
| pH | T5 | 0.4811 |
| pH | T6 | 0.5852 |
| pH | T7 | 0.2103 |
| pCO2 | T1 | 0.7499 |
| pCO2 | T2 | 0.5259 |
| pCO2 | T3 | 0.4100 |
| pCO2 | T4 | 0.6650 |
| pCO2 | T5 | 0.7645 |
| pCO2 | T6 | 0.1601 |
| pCO2 | T7 | 0.2742 |
| BD | T1 | 0.3104 |
| BD | T2 | 0.1287 |
| BD | T3 | 0.3935 |
| BD | T4 | 0.0845 |
| BD | T5 | 0.9152 |
| BD | T6 | 0.2639 |
| BD | T7 | 0.0058 |
| Lactate | T1 | 0.6974 |
| Lactate | T2 | 0.0337 |
| Lactate | T3 | 0.0284 |
| Lactate | T4 | 0.3911 |
| Lactate | T5 | 0.0134 |
| Lactate | T6 | 0.0239 |
| Lactate | T7 | 0.6862 |
| PTT | T1 | 0.2173 |
| PTT | T2 | 0.8003 |
| PTT | T3 | 0.8107 |
| PTT | T4 | 0.7833 |
| PTT | T5 | 0.5853 |
| PTT | T6 | 0.5366 |
| PTT | T7 | 0.4096 |
| d.dimer | T1 | 0.2168 |
| d.dimer | T2 | 0.0404 |
| d.dimer | T3 | 0.5271 |
| d.dimer | T4 | 0.1218 |
| d.dimer | T5 | 0.6370 |
| d.dimer | T6 | 0.8792 |
| d.dimer | T7 | 0.0201 |
| PT-INR | T1 | 0.3127 |
| PT-INR | T2 | 0.8118 |
| PT-INR | T3 | 0.1932 |
| PT-INR | T4 | 0.0116 |
| PT-INR | T5 | 0.5354 |
| PT-INR | T6 | 0.1882 |
| PT-INR | T7 | 0.9122 |
| Fibrinogen | T1 | 0.7344 |
| Fibrinogen | T2 | 0.1494 |
| Fibrinogen | T3 | 0.8705 |
| Fibrinogen | T4 | 0.6466 |
| Fibrinogen | T5 | 0.8278 |
| Fibrinogen | T6 | 0.2435 |
| Fibrinogen | T7 | 0.5171 |
| WBC | T1 | 0.8269 |
| WBC | T2 | 0.7837 |
| WBC | T3 | 0.7976 |
| WBC | T4 | 0.7547 |
| WBC | T5 | 0.4293 |
| WBC | T6 | 0.9003 |
| WBC | T7 | 0.8639 |
| Platelet | T1 | 0.0585 |
| Platelet | T2 | 0.8943 |
| Platelet | T3 | 0.4924 |
| Platelet | T4 | 0.8643 |
| Platelet | T5 | 0.3788 |
| Platelet | T6 | 0.5207 |
| Platelet | T7 | 0.3944 |
| CK | T1 | 0.2292 |
| CK | T2 | 0.2702 |
| CK | T3 | 0.6584 |
| CK | T5 | 0.5416 |
| Glucose | T1 | 0.8111 |
| Glucose | T2 | 0.6537 |
| Glucose | T3 | 0.1433 |
| Glucose | T4 | 0.2828 |
| Glucose | T5 | 0.2466 |
| Glucose | T6 | 0.0126 |
| Glucose | T7 | 0.0151 |
| Cortisol | T2 | 0.8709 |
| Cortisol | T3 | 0.1144 |
| Cortisol | T4 | 0.4740 |
| Cortisol | T5 | 0.3373 |
| Cortisol | T6 | 0.1384 |
| Cortisol | T7 | 0.4993 |
| Creatinine | T1 | 0.7933 |
| Creatinine | T2 | 0.5927 |
| Creatinine | T3 | 0.6180 |
| Creatinine | T4 | 0.7113 |
| Creatinine | T5 | 0.5044 |
| Creatinine | T6 | 0.6793 |
| Creatinine | T7 | 0.6009 |

Caption: P-values for the associations between biomarker concentrations at each timepoint and Cerebellar injury severity.
