# Supplementary material for: Lab values in neonates with hypoxic ischemic encephalopathy over time during and after therapeutic hypothermia
Source: Front Pediatr. 2026 Mar 12;14:1743749. doi: 10.3389/fped.2026.1743749 (PMC13017858; doi:10.3389/fped.2026.1743749)
Supplement: Supplementary file 5 [file Table5.docx]

**Supplementary Table 5.** Associations between systemic biomarkers and Total Brain MRI injury scores across timepoints.

| Biomarker | Timepoint | p-value |
| --- | --- | --- |
| ALT | T1 | 0.4431 |
| ALT | T2 | 0.0002 |
| ALT | T3 | 0.0243 |
| ALT | T4 | 0.0011 |
| ALT | T5 | 0.0302 |
| ALT | T6 | 0.3925 |
| ALT | T7 | 0.3660 |
| AST | T1 | 0.3104 |
| AST | T2 | 0.0005 |
| AST | T3 | 0.7038 |
| AST | T4 | 0.0070 |
| AST | T5 | 0.2981 |
| AST | T6 | 0.9551 |
| AST | T7 | 0.3425 |
| Bilirubin | T1 | 0.0746 |
| Bilirubin | T2 | 0.0028 |
| Bilirubin | T3 | 0.0661 |
| Bilirubin | T4 | 0.4024 |
| Bilirubin | T5 | 0.0650 |
| Bilirubin | T6 | 0.0983 |
| Bilirubin | T7 | 0.9033 |
| pH | T1 | 0.0106 |
| pH | T2 | 0.0454 |
| pH | T3 | 0.0764 |
| pH | T4 | 0.2336 |
| pH | T5 | 0.6870 |
| pH | T6 | 0.9458 |
| pH | T7 | 0.4397 |
| pCO2 | T1 | 0.0007 |
| pCO2 | T2 | 0.0354 |
| pCO2 | T3 | 0.5663 |
| pCO2 | T4 | 0.3704 |
| pCO2 | T5 | 0.2567 |
| pCO2 | T6 | 0.4729 |
| pCO2 | T7 | 0.9590 |
| BD | T1 | <0.0001 |
| BD | T2 | <0.0001 |
| BD | T3 | 0.0163 |
| BD | T4 | 0.0022 |
| BD | T5 | 0.5391 |
| BD | T6 | 0.4877 |
| BD | T7 | 0.2437 |
| Lactate | T1 | <0.0001 |
| Lactate | T2 | <0.0001 |
| Lactate | T3 | <0.0001 |
| Lactate | T4 | <0.0001 |
| Lactate | T5 | <0.0001 |
| Lactate | T6 | <0.0001 |
| Lactate | T7 | 0.0222 |
| PTT | T1 | 0.0030 |
| PTT | T2 | 0.4239 |
| PTT | T3 | 0.2882 |
| PTT | T4 | 0.1048 |
| PTT | T5 | 0.0776 |
| PTT | T6 | 0.2119 |
| PTT | T7 | 0.8687 |
| d.dimer | T1 | 0.0085 |
| d.dimer | T2 | 0.2873 |
| d.dimer | T3 | 0.0003 |
| d.dimer | T4 | 0.0065 |
| d.dimer | T5 | 0.0482 |
| d.dimer | T6 | 0.8934 |
| d.dimer | T7 | 0.8569 |
| PT-INR | T1 | <0.0001 |
| PT-INR | T2 | 0.3385 |
| PT-INR | T3 | 0.0007 |
| PT-INR | T4 | 0.7120 |
| PT-INR | T5 | 0.6629 |
| PT-INR | T6 | 0.6581 |
| PT-INR | T7 | 0.6937 |
| Fibrinogen | T1 | 0.1362 |
| Fibrinogen | T2 | 0.4242 |
| Fibrinogen | T3 | 0.4098 |
| Fibrinogen | T4 | 0.5015 |
| Fibrinogen | T5 | 0.0128 |
| Fibrinogen | T6 | 0.4322 |
| Fibrinogen | T7 | 0.2441 |
| WBC | T1 | 0.0902 |
| WBC | T2 | 0.7435 |
| WBC | T3 | 0.0935 |
| WBC | T4 | 0.8765 |
| WBC | T5 | 0.5559 |
| WBC | T6 | 0.9964 |
| WBC | T7 | 0.7317 |
| Platelet | T1 | 0.8298 |
| Platelet | T2 | 0.0556 |
| Platelet | T3 | 0.2393 |
| Platelet | T4 | 0.4213 |
| Platelet | T5 | 0.0185 |
| Platelet | T6 | 0.0067 |
| Platelet | T7 | 0.0051 |
| CK | T1 | 0.3652 |
| CK | T2 | 0.7203 |
| CK | T3 | 0.3751 |
| CK | T4 | 0.5826 |
| CK | T5 | 0.4127 |
| CK | T6 | 0.5326 |
| CK | T7 | 0.6327 |
| Glucose | T1 | 0.0170 |
| Glucose | T2 | 0.0469 |
| Glucose | T3 | 0.3899 |
| Glucose | T4 | 0.0209 |
| Glucose | T5 | <0.0001 |
| Glucose | T6 | 0.0004 |
| Glucose | T7 | 0.0018 |
| Cortisol | T1 | 0.6799 |
| Cortisol | T2 | 0.7373 |
| Cortisol | T3 | 0.8407 |
| Cortisol | T4 | 0.2313 |
| Cortisol | T5 | 0.6998 |
| Cortisol | T6 | 0.0276 |
| Cortisol | T7 | 0.6208 |
| Creatinine | T1 | 0.8085 |
| Creatinine | T2 | 0.6375 |
| Creatinine | T3 | 0.6883 |
| Creatinine | T4 | 0.7628 |
| Creatinine | T5 | 0.8659 |
| Creatinine | T6 | 0.6840 |
| Creatinine | T7 | 0.7872 |

Caption: P-values for the associations between biomarker concentrations at each timepoint and overall brain injury severity.
